# Progressive Prefrontal Cortex Dysfunction in Parkinson's Disease With Probable REM Sleep Behavior Disorder: A 3-Year Longitudinal Study

**DOI:** 10.3389/fnagi.2021.750767

**Published:** 2022-01-10

**Authors:** Xiuqin Jia, Wentao Fan, Zhijiang Wang, Yuehong Liu, Ying Li, Haibin Li, Hui Li, Ting Ma, Jing Wang, Qi Yang

**Affiliations:** ^1^Department of Radiology, Beijing Chaoyang Hospital, Capital Medical University, Beijing, China; ^2^Key Lab of Medical Engineering for Cardiovascular Disease, Ministry of Education, Beijing, China; ^3^Beijing Advanced Innovation Center for Big Data-Based Precision Medicine, Beijing, China; ^4^Department of Radiology, Beijing Geriatric Hospital, Beijing, China; ^5^National Clinical Research Center for Mental Disorders, Peking University Sixth Hospital (Institute of Mental Health), Beijing, China; ^6^Department of Radiology, Beijing Anzhen Hospital, Capital Medical University, Beijing, China; ^7^Department of Cardiac Surgery, Heart Center, Beijing Chaoyang Hospital, Capital Medical University, Beijing, China; ^8^School of Electronic and Information Engineering, Harbin Institute of Technology at Shenzhen, Shenzhen, China; ^9^Department of Clinical Lab, Beijing Chaoyang Hospital, Capital Medical University, Beijing, China

**Keywords:** frontopolar cortex, dorsolateral prefrontal cortex, RBD, functional connectivity, Parkinson's disease

## Abstract

This study aimed to explore the disrupted prefrontal cortex activity specific to patients with Parkinson's disease (PD) with rapid eye movement sleep behavior disorder (RBD) compared with those without and to further examine the associations between these alterations and neuropsychological measurements. Ninety-six patients with early PD underwent both structural and functional MRI, and also neuropsychological assessments in the Parkinson's Progression Markers Initiative (PPMI) database. Of these, 46 patients who completed 1- and 3-year fMRI follow-up examinations were categorized as PD with probable RBD (PD-pRBD^+^) and without (PD-pRBD^−^). The left dorsolateral prefrontal cortex (DLPFC) seed-to-voxel functional connectivity analysis was conducted to evaluate the progressive neural alterations specific to PD-pRBD^+^ compared with PD-pRBD^−^ over time. Furthermore, relationships between these alterations and neuropsychological performance were examined. Compared with patients with PD-pRBD^−^, patients with PD-pRBD^+^ initially exhibited connectivity deficits between the left DLPFC and the medial frontopolar cortex. Moreover, these patients further exhibited disrupted DLPFC connectivity in the lateral frontopolar cortex at the 3-year follow-up evaluation. Correlation analysis revealed that connectivity between the left DLPFC and frontopolar cortex was positively related to executive function in PD-pRBD^+^ after adjusting for nuisance variables. Progressive prefrontal cortex dysfunction associated with RBD in early PD may provide an effective subtype-specific biomarker of neurodegenerative progression, which may shed light on the neuropathological mechanisms underlying the clinical heterogeneity of this disease.

## Introduction

Sleep disturbances are frequently experienced in patients with Parkinson's disease (PD) (Fereshtehnejad et al., 2017; Jozwiak et al., 2017). Specifically, rapid eye movement (REM) sleep behavior disorder (RBD) affects 30–40% of PD (Sixel-Döring et al., 2016), which is associated with early neuropathological progression of this disease (Muzur et al., 2002; Vendette et al., 2007; Kim and Jeon, 2014; Pagano et al., 2018; Kim et al., 2019, 2020; Figorilli et al., 2020). However, the pathophysiology of RBD in PD is still widely unknown.

Currently, evidence has emerged linking RBD in PD to prefrontal cortex dysfunction (Lerche and Brockmann, 2018; Hanuška et al., 2019). A growing body of evidence have revealed that patients with PD having RBD have significantly impaired executive function (Fantini et al., 2018; Kamble et al., 2019), which significantly worsened during the progression of RBD symptoms in PD (Figorilli et al., 2020). In particular, a body of task functional magnetic resonance imaging (fMRI) studies have demonstrated that the dorsolateral prefrontal cortex (DLPFC) plays a cardinal role in executive function (Perlstein et al., 2001; Jia et al., 2011, 2015b; Liang et al., 2014, 2016; Panikratova et al., 2020). Furthermore, the DLPFC has been implicated in both REM-related cortical activation (Ioannides et al., 2009) and the pathophysiological basis of neurodegenerative progression in PD (Doruk et al., 2014). However, little is known about whether DLPFC activity demonstrates a specific alteration pattern in patients with PD having RBD compared with those without RBD. The neurobiological mechanisms underlying this dysfunction in early PD and the progressive alterations over time remain largely unexplored.

Accordingly, the progressive functional alterations specific to patients with PD with RBD were of particular interest in this work. The aims of this work were to explore the altered functional connectivity (FC) in the DLPFC specific to PD in patients with probable RBD (PD-pRBD^+^) compared with those in patients without probable RBD (PD-pRBD^−^) and to further explore the associations between these alterations and neuropsychological measures in these patients. On the basis of previous studies (Ioannides et al., 2009; Doruk et al., 2014; Panikratova et al., 2020), it was hypothesized that patients with PD-pRBD^+^ might exhibit prefrontal cortex dysfunction during the progression of this disease.

## Materials and Methods

### Participants

Participants were acquired from Parkinson's Progression Markers Initiative (PPMI) (http://www.ppmi-info.org/). The inclusion criteria were as follows: (1) at least two motor symptoms, (2) PD diagnosis within 2 years at baseline and an early clinical disease stage with Hoehn and Yahr stage of I–II, (3) no symptomatic treatment within 6 months of baseline, and (4) a dopamine transporter (DAT) deficit.

In this work, only individuals who underwent both structural and fMRI scanning were included. In total, there were 96 patients with PD and 22 healthy controls (HCs) at the acquisition of the first fMRI scan (i.e., baseline fMRI), 80 patients with PD at the 1-year follow-up, and 46 patients with PD at the 3-year follow-up. The study was approved by the institutional review board at each PPMI site. Written informed consent from all participants was obtained.

### Clinical and Neuropsychological Assessments

For each participant, a comprehensive set of motor and non-motor symptoms was assessed. The severity and stage of the disease were assessed by the Movement Disorder Society-Unified Parkinson's Disease Rating Scale III (MDS-UPDRS III) and the Hoehn and Yahr stage. Sleep disturbance was evaluated by the Epworth sleepiness scale (ESS) and RBD screening questionnaire (RBDSQ).

**Figure 1:**
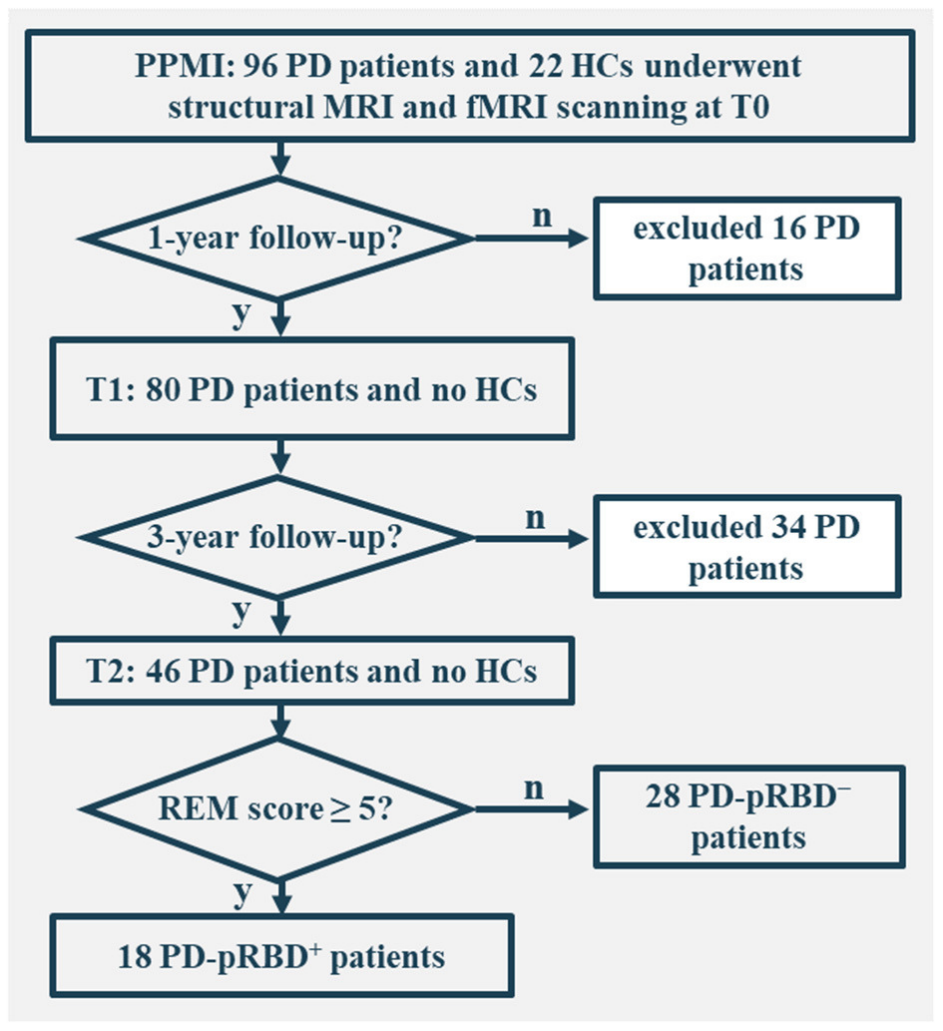
Flowchart of data inclusion. T0, fMRI data at onset; T1, the 1-year follow-up evaluation; T2, the 3-year follow-up evaluation; PD-pRBD^+^, Parkinson's disease (PD) with probable rapid eye movement (REM) sleep behavior disorder (pRBD); PD-pRBD^−^, PD without pRBD; HC, healthy control.

All participants were administered the Montreal Cognitive Assessment (MoCA) to evaluate global cognition, the Benton Judgment of Line Orientation Score (JLO) to assess visuospatial ability, the Hopkins Verbal Learning Test-Revised (HVLT-R) to evaluate memory, the Letter–Number Sequencing (LNS), and Semantic Fluency to assess executive function/working memory, and the Symbol Digit Modalities Test (SDMT) to evaluate attention. Autonomic symptoms were assessed by the Scales for Outcomes in PD-Autonomic (SCOPA-AUT), depression was assessed by the 15-item Geriatric Depression Scale (GDS-15), and anxiety was evaluated by the State-Trait Anxiety Inventory (STAI) score. For the purpose of longitudinal assessments, the clinical and MRI data of the 46 patients with PD who completed all 3 fMRI visits (i.e., onset, 1-, and 3**-**year follow-up) were comprehensively analyzed. Of these, 18 patients with PD were categorized as PD-pRBD^+^ and 28 as PD-pRBD^−^ according to RBDSQ with a cut-off score of 5, as suggested by the PPMI database (Mahajan et al., 2014) (Figure 1). In addition, the data of HCs were also included for comparison with the data of the patients. Clinical characteristics and neuropsychological assessments are presented in Table 1.

**Table 1:** Longitudinal demographic characteristics and neuropsychological assessments of PD-pRBD^+^ and PD-pRBD^−^ patients over time.

	**PD-pRBD^+^ (*****n*** **=** **18)**			**PD-pRBD^−^ (*****n*** **=** **28)**			***P*** **_between-group_**		
	**T0**	**T1**	**T2**	**T0**	**T1**	**T2**	**T0**	**T1**	**T2**
Age (years)	61.72 (11.24)	62.78 (11.24)	64.89 (11.10)	60.29 (8.58)	61.18 (8.49)	63.11 (8.52)	0.614	0.586	0.542
Sex (male/female)	14/4	14/4	14/4	16/12	16/12	16/12	0.152	0.152	0.152
Education (years)	16.17 (3.09)	16.17 (3.09)	16.17 (3.09)	14.75 (3.57)	14.75 (3.57)	14.75 (3.57)	0.174	0.174	0.174
Hoehn and Yahr stage	1.57 (0.53)	1.89 (0.33)	2.11 (0.32)	1.70 (0.48)	1.79 (0.40)	1.68 (0.48)	0.612	0.56	**0.002**
MDS-UPDRS part III	18.67 (10.48)	20.56 (10.45)	23.61 (15.37)	18.61 (9.62)	20.18 (9.24)	23.96 (10.95)	0.943	0.967	0.928
Disease duration (months)	17.94 (11.53)	27.28 (9.68)	43.06 (9.68)	18.25 (9.02)	27.11 (7.76)	42.82 (8.26)	0.920	0.948	0.931
LEDD (mg/day)	186.78 (212.01)	387.20 (262.82)	508.26 (286.55)	96.82 (140.69)	291.31 (241.85)	389.94 (261.66)	0.135	0.158	0.156
GDS	2.06 (1.59)	2.06 (2.04)	1.94 (2.01)	1.86 (2.53)	2.07 (2.14)	2.00 (2.13)	0.832	0.97	0.93
STAI	68.39 (17.79)	66.61 (17.40)	61.89 (16.26)	62.96 (18.67)	66.39 (20.22)	62.75 (17.82)	0.333	0.98	0.869
MoCA	26.00 (3.58)	26.56 (3.18)	26.44 (4.12)	28.04 (1.95)	28.18 (1.79)	28.39 (2.15)	**0.016**	**0.032**	**0.041**
JLO	12.18 (2.31)	12.24 (2.95)	12.12 (2.74)	12.12 (2.75)	12.47 (2.66)	12.43 (3.00)	0.931	0.79	0.728
HVLT-R total recall	45.06 (12.62)	46.22 (13.28)	48.06 (10.69)	49.86 (13.00)	50.04 (12.00)	51.11 (11.90)	0.223	0.318	0.382
HVLT-R recognition	42.78 (13.97)	44.67 (12.90)	50.33 (8.74)	47.82 (11.70)	48.46 (10.23)	48.29 (8.78)	0.193	0.274	0.444
LNS	10.78 (2.44)	10.56 (3.01)	10.89 (2.59)	12.32 (2.14)	13.04 (2.22)	12.61 (2.94)	**0.029**	**0.002**	**0.044**
SFT	55.28 (10.19)	52.39 (10.59)	53.56 (11.79)	57.32 (9.99)	56.46 (8.81)	55.46 (11.11)	0.505	0.164	0.582
SDMT	43.02 (8.99)	43.46 (10.21)	42.60 (10.96)	48.37 (9.06)	47.72 (7.38)	47.71 (8.38)	0.056	0.108	0.081
ESS	7.50 (4.91)	8.00 (4.24)	9.28 (5.68)	4.86 (3.73)	5.00 (3.95)	5.64 (4.67)	**0.044**	**0.019**	**0.022**
RBDSQ	5.33 (2.66)	6.11 (2.27)	8.11 (2.30)	2.43 (1.26)	2.50 (1.35)	2.14 (1.18)	**<0.001**	**<0.001**	**<0.001**
SCOPA-AUT	9.44 (6.01)	11.00 (7.81)	11.39 (5.21)	8.79 (6.27)	9.04 (4.84)	10.04 (5.24)	0.726	0.297	0.396
Lt.Caudate SBR	1.61 (0.60)	1.43 (0.47)	1.31 (0.61)	1.95 (0.48)	1.77 (0.54)	1.68 (0.48)	**0.040**	**0.033**	**0.028**
Lt.Putamen SBR	0.58 (0.22)	0.51 (0.16)	0.45 (0.22)	0.78 (0.31)	0.70 (0.24)	0.65 (0.27)	**0.024**	**0.003**	**0.011**
Rt.Caudate SBR	1.73 (0.54)	1.54 (0.48)	1.41 (0.60)	1.95 (0.45)	1.79 (0.54)	1.68 (0.47)	0.131	0.117	0.085
Rt.Putamen SBR	0.68 (0.27)	0.67 (0.27)	0.49 (0.25)	0.79 (0.30)	0.70 (0.27)	0.67 (0.22)	0.206	0.661	**0.012**

*Data are expressed as the mean (standard deviation). Sex data were analyzed with a χ^2^-test. Other p-values were derived from a Mann-Whitney U-test for non-parametric tests or two sample t-tests for parametric data. T0, the onset of fMRI data; T1, the 1-year follow-up evaluaction; T2, the 3-year follow-up evaluation; PD-pRBD^+^, Parkinson's disease (PD) with probable rapid eye movement (REM) sleep behavior disorder (pRBD); PD-pRBD^−^, PD without pRBD; MDS-UPDRS, Movement Disorder Society-Unified Parkinson's Disease Rating Scale; LEDD, levodopa equivalent daily dose; GDS, Geriatric Depression Scale; STAI, State-Trait Anxiety Index; MoCA, Montreal Cognitive Assessment; JLO, Benton Judgment of Line Orientation; HVLT-R, Hopkins Verbal Learning Test-Revised; SFT, semantic fluency test; SDMT, Symbol Digit Modalities Test; LNS, Letter-Number Sequencing; SFT, semantic fluency test; SDMT, symbol digit modalities test; ESS, Epworth Sleepiness Scale; RBDSQ, RBD Screening Questionnaire; SCOPA-AUT, Scales for Outcomes in Parkinson's disease—Autonomic; SBR, striatal binding ratio; Lt, left; Rt, right*.

*Bold values mean p < 0.05*.

### MRI Data Acquisition

High-resolution structural images were acquired by using 3D T1-weighted MPRAGE. The scan parameters were as follows: repetition time (TR) = 2,300 ms, echo time (TE) = 2.89 ms, flip angle = 9°, matrix size = 256 × 240, and 176 1 mm sagittal slices. FMRI data were obtained by using an EPI sequence that lasted 7 min (210 volumes) with the following parameters: TR = 2,400 ms, TE = 25 ms, flip angle = 80°, field of view = 240 mm × 240 mm, matrix size = 68 × 66, 40 slices, and slice thickness = 3.3 mm. Since fMRI data were not administered at first in beginning of the PPMI project, in this work, the baseline fMRI referred to first fMRI examination performed upon onset.

### MRI Data Preprocessing

#### Voxel-Based Morphometry Analysis

We performed a voxel-based morphometry (VBM) analysis using SPM12 (http://www.fil.ion.ucl.ac.uk/spm) during the visit of patients with PD to determine structural alterations between the two groups during disease progression. T1-weighted images were segmented using the unified segmentation model into gray matter (GM), white matter (WM), and cerebrospinal fluid (CSF) based on tissue probability maps in Montreal Neurological Institute (MNI) space. The spatially normalized GM maps were modulated by the Jacobian determinant of the deformation field and corrected for individual brain sizes. The modulated and normalized GM images (voxel size: 1.5 × 1.5 × 1.5 mm^3^) were smoothed with an 8-mm full width at half maximum (FWHM) isotropic Gaussian kernel.

#### Rs-fMRI Preprocessing and Functional Connectivity

Rs-fMRI data were preprocessed using SPM12, and seed-to-voxel correlation analysis was carried out by the FC (CONN) toolbox v19c (Whitfield-Gabrieli and Nieto-Castanon, 2012). The first 10 functional images were discarded to reduce the fluctuation of the MRI signal in the initial stage of scanning. The remaining 200 images of each participant were first corrected for slice timing and then realigned to eliminate the influence of head motion. Next, the realigned images were coregistered to T1 images and normalized into MNI template space using transformations from segmentation and resampled to 3 × 3 × 3 mm^3^. Subsequently, images were smoothed with a 6-mm FWHM isotropic Gaussian kernel.

After preprocessing, the images were then bandpass-filtered to 0.008–0.09 Hz to remove physiological noise. Artifact detection tools-based identification of outlier scans for scrubbing was performed, after which more than 90% of the valid scans remained. Further denoising steps included regression of the six motion parameters and their first-order derivatives, regression of WM and CSF signals following the implemented CompCor strategy (Behzadi et al., 2007), and linear detrending.

The seed region of the left DLPFC was defined by WFU_PickAtlas (http://fmri.wfubmc.edu/cms/software). The correlation coefficients between the seed voxels and all other brain voxels were computed to generate correlation maps. For group analyses, the correlation coefficients were converted to *z*-values using Fisher's *r*-to-*z* transformation to improve normality (Lowe et al., 1998).

### Statistical Analysis

For clinical and neuropsychological measurements, the normality of clinical data and neuropsychological measures was evaluated by a Kolmogorov–Smirnov test to choose parametric or non-parametric tests using SPSS v22 (Armonk, NY: IBM Corp). General linear models were performed on both GM maps and FC maps using a voxelwise comparison across the whole brain. An absolute GM threshold of 0.2 was used to avoid possible edge effects around the border between GM and WM. Mixed repeated measures analysis of variance was performed to compare the longitudinal changes between PD groups with visit-time (i.e., T0, T1, T2) as within-group factor and group (i.e., PD-pRBD^+^ and PD-pRBD^−^) as between-group factor. *Post-hoc* comparisons were further performed to determine longitudinal changes over time within group and differences between group at each visit time. FC results were reported based on an uncorrected voxelwise height threshold of *p* < 0.001 combined with an FWE-corrected clusterwise threshold of *p* < 0.05. Brain regions were localized based on Anatomy toolbox v2.2c (Eickhoff et al., 2005).

Regions with significant alterations in patients with PD-pRBD^+^ were further defined as regions of interest (ROIs). Aging is a risk factor in PD-pRBD^+^ (Dauvilliers et al., 2018). In this work, age was taken as a nuisance variable between group comparisons to control its confounding effect. Furthermore, to explore the relationship between FC values in these ROIs and performance of the patients on neuropsychological assessments, partial correlations were performed after adjusting for age, sex, education level, disease duration, and levodopa dose as nuisance variables of no interest.

## Results

### Demographic Characteristics

As listed in Table 1, significantly increased disease stage, levodopa dose, and severe sleep disturbance were found in patients with PD-pRBD^+^ over time, as measured by Hoehn and Yahr stage (*p* = 0.010), levodopa equivalent daily dose (LEDD) (*p* = 0.002), and RBDSQ (*p* = 0.003), respectively. *Post-hoc* comparisons revealed that significantly higher Hoehn and Yahr stages (*p* < 0.05) and LEDD (*p* < 0.05) were detected at the 3-year follow-up evaluation compared with baseline in patients with PD-pRBD^+^. Regarding sleep behavior, significantly higher RBDSQ scores were identified at the 3-year follow-up evaluation than at both the 1-year follow-up (*p* < 0.05) and baseline (*p* < 0.05) in patients with PD-pRBD^+^. For patients with PD-pRBD^−^, a significantly higher LEDD was found over time (*p* < 0.001), with significantly higher LEDDs at the 1- and 3-year follow-up evaluations than at baseline (*p* < 0.05).

**Figure 2:**
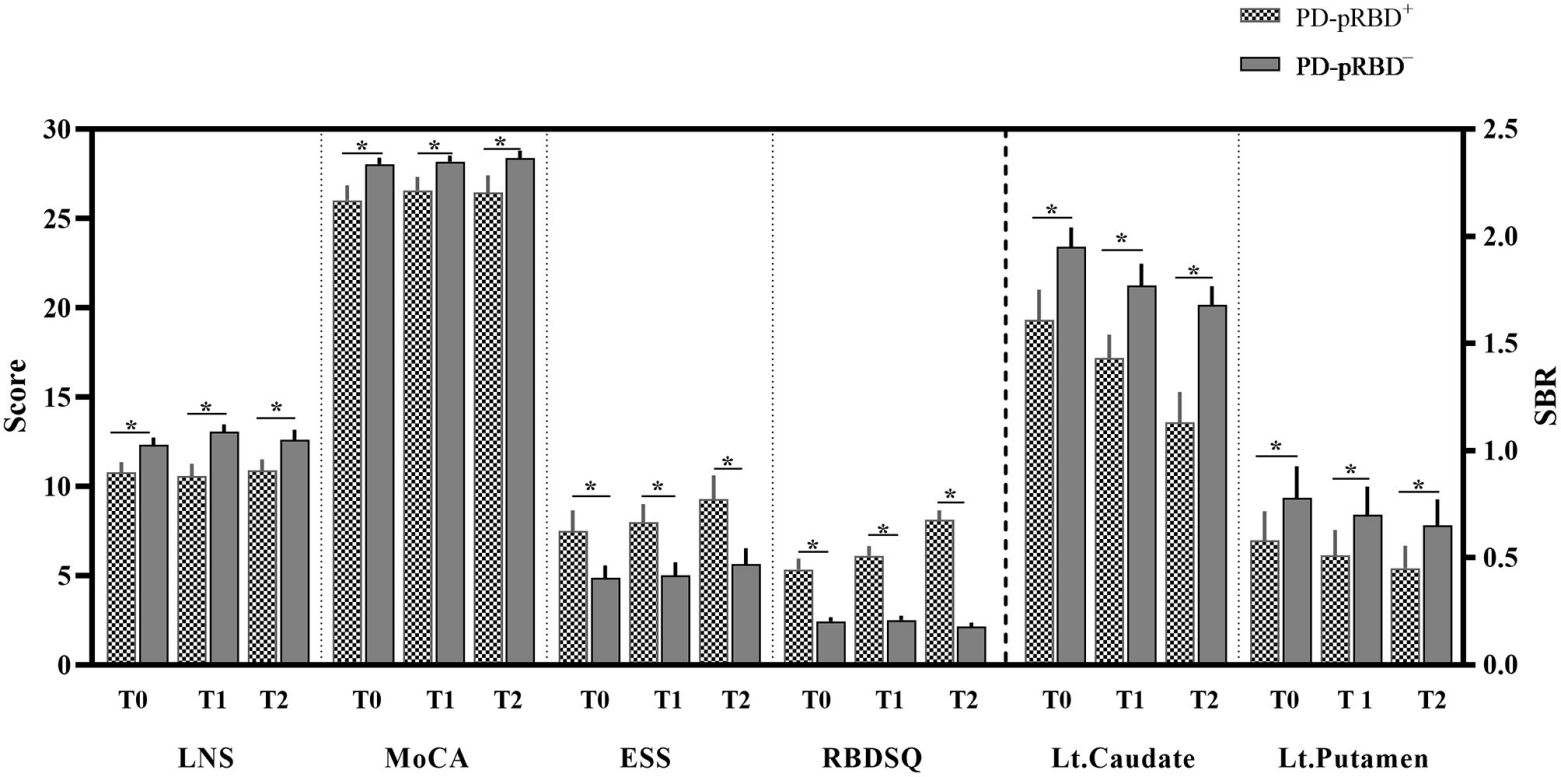
Significantly different neuropsychological assessments between-group comparisons. PD-pRBD^+^, Parkinson's disease (PD) with probable rapid eye movement (REM) sleep behavior disorder (pRBD); PD-pRBD^−^, PD without pRBD; T0, fMRI data at onset; T1, the 1-year follow-up evaluation; T2, the 3-year follow-up evaluation; LNS, letter-number sequencing; MoCA, Montreal cognitive assessment; ESS, Epworth sleepiness scale; RBDSQ, RBD screening questionnaire; SBR, striatal binding ratio; Lt, left; *, showed significant differences between patients with PD-pRBD^+^ and PD-pRBD^−^.

As shown in Figure 2, regarding between-group comparisons, significantly decreased general cognition measured by MoCA scores were revealed in patients with PD-pRBD^+^ compared with PD-pRBD^−^, particularly, at T0 with (26.00 ± 3.58) vs. (28.04 ± 1.95), *p* = 0.016, at T1 with (26.56 ± 3.18) vs. (28.18 ± 1.79), *p* = 0.032, and at T2 with (26.44 ± 4.12) vs. (28.39 ± 2.15), *p* = 0.04, respectively. In addition, in contrast to patients with PD-pRBD^−^, patients with PD-pRBD^+^ exhibited significant deficit in executive function measured by LNS scores at T0 with (10.78 ± 2.44) vs. (12.32 ± 2.14), *p* = 0.029; at T1 with (10.56 ± 3.01) vs. (13.04 ± 2.22), *p* = 0.002; and at T2 with (10.89 ± 2.59) vs. (12.61 ± 2.94), *p* = 0.044, respectively. Compared with patients with PD-pRBD^−^, the left caudate and putamen striatal binding ratios (SBRs) revealed significant decrease in patients with PD-pRBD^+^ at each visit (all *p* < 0.05). In addition, a significant decrease in the right putamen SBR was found in patients with PD-pRBD^+^ compared with PD-pRBD^−^ patients at the 3-year follow-up evaluation (*p* < 0.05). No significant differences were detected in the remaining comparisons.

### VBM Results

**Figure 3:**
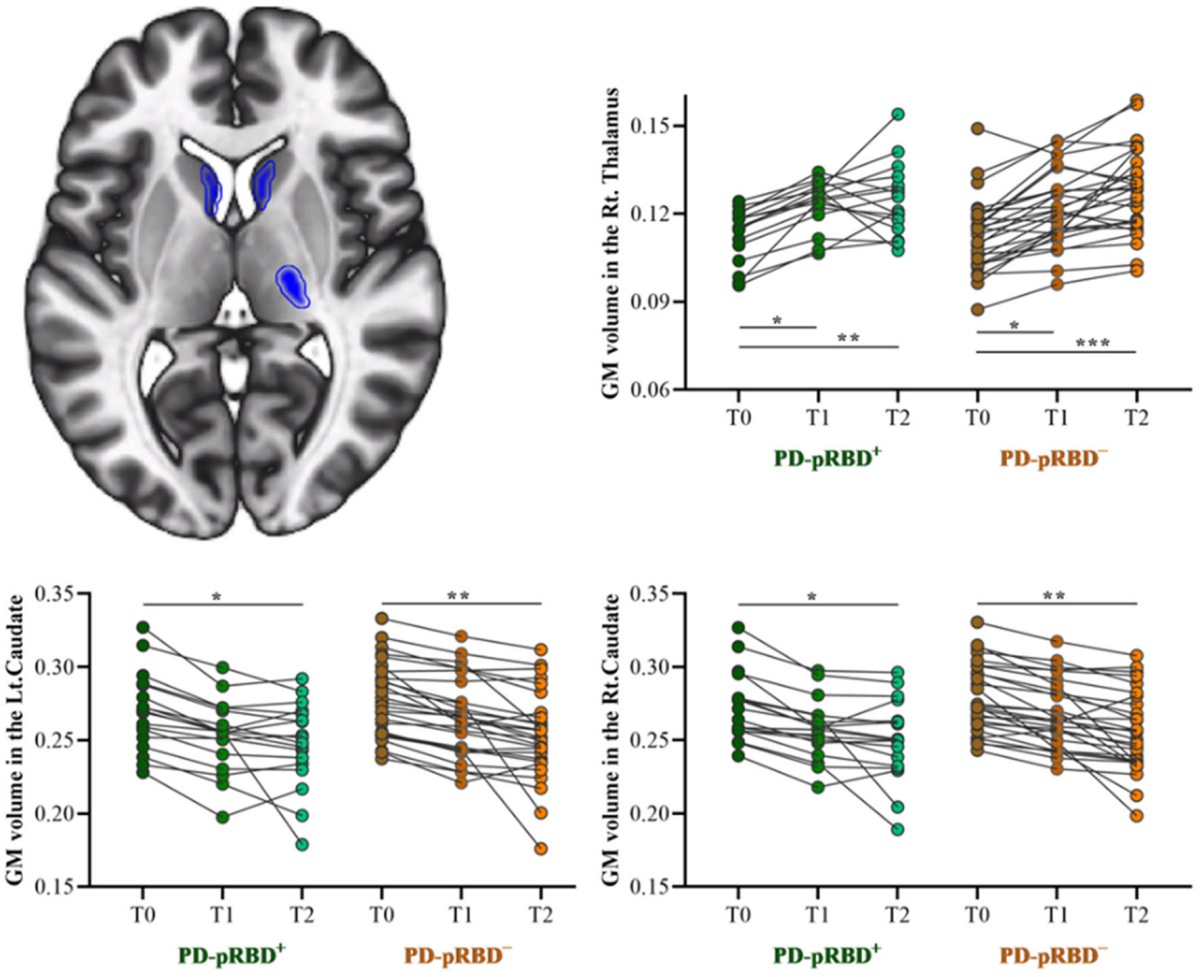
Longitudinal GM volume changes in patients with both PD-pRBD^+^ and PD-pRBD^−^ over time. The results were thresholded based on a voxelwise height threshold of *p* < 0.05 (FWE-corrected). Mixed repeated measures analysis of variance was performed to compare the longitudinal changes between PD groups with *visit-time* (i.e., T0, T1, T2) as within-group factor and *group* (i.e., PD-pRBD^+^ and PD-pRBD^−^) as between-group factor. *Post-hoc* comparisons were further performed to determine longitudinal changes over time within group and differences between groups at each visit time. PD-pRBD^+^, Parkinson's disease (PD) with probable rapid eye movement (REM) sleep behavior disorder (pRBD); PD-pRBD^−^, PD without pRBD; GMV, gray matter volume; Lt, left; Rt, right. *p < 0.05; **p < 0.01; ***p < 0.001.

No significant crosssectional GM volume difference was detected between PD-pRBD^+^ and PD-pRBD^−^ groups. Longitudinally, it was found that significantly decreased GM volume in the bilateral caudate (MNI: −9, 2, 14, *F* = 19.75, 365 voxels, and MNI: 11, 3, 17, *F* = 28.09, 466 voxels, respectively) and increased GM volume in the right thalamus (MNI: 23, −21, 6; *F* = 29.96, 315 voxels) were conjunctively detected in patients with PD over time (Figure 3), which was consistent with our previous findings (Jia et al., 2015a).

### Functional Connectivity Results

**Figure 4:**
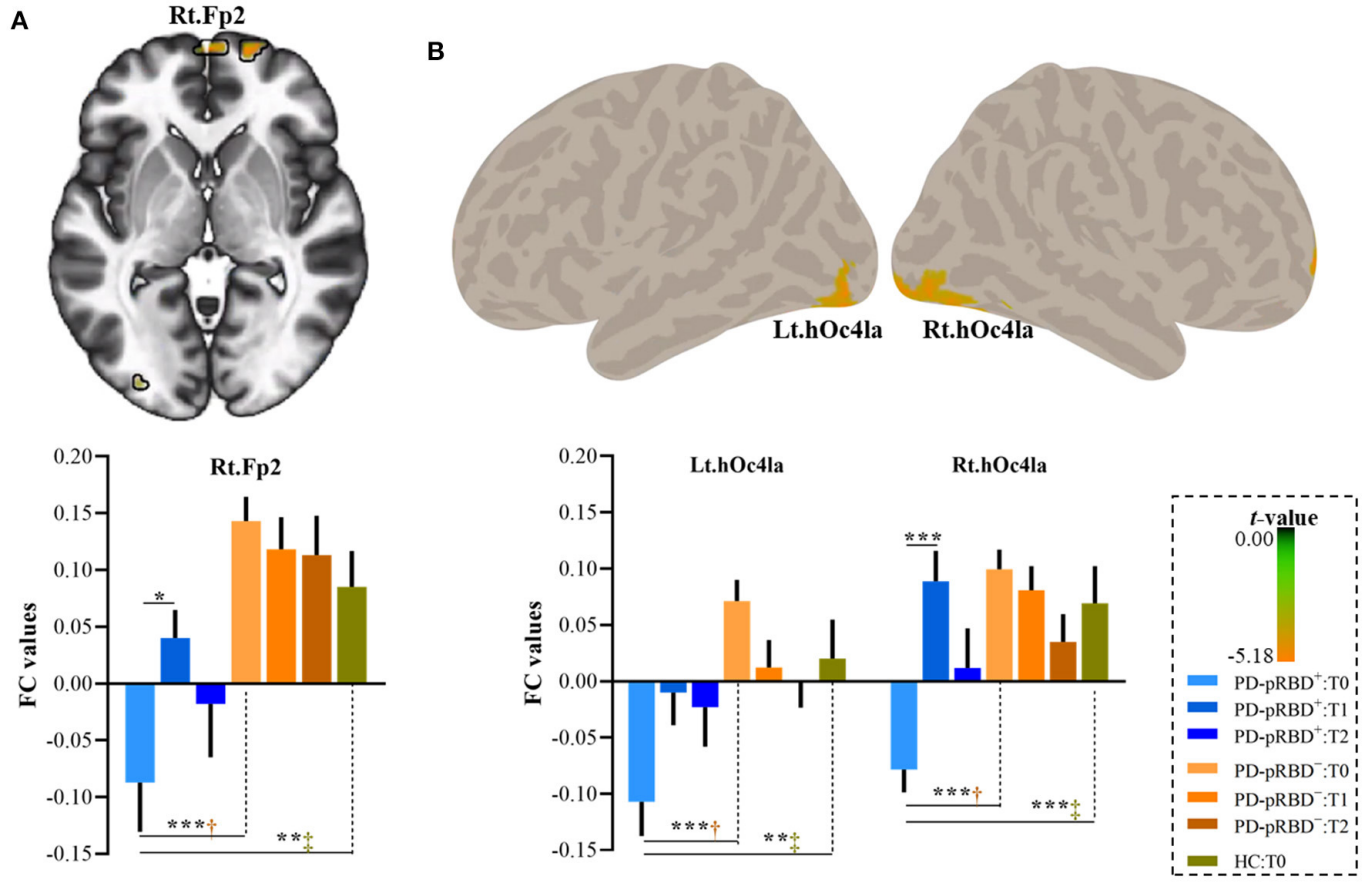
Decreased FC in the DLPFC in PD-pRBD^+^ compared to PD-pRBD^−^ at baseline fMRI scans. **(A)** Decreased FC in the right Fp2; **(B)** Decreased FC in the bilateral hOc4la. Bar graph showed the mean extracted FC values in these regions. The results were thresholded based on an uncorrected voxelwise height threshold of *p* < 0.001 combined with an FWE-corrected clusterwise threshold of *p* < 0.05. Mixed repeated measures analysis of variance was performed to compare the longitudinal changes between PD groups with *visit-time* (i.e., T0, T1, T2) as within-group factor and *group* (i.e., PD-pRBD^+^ and PD-pRBD^−^) as between-group factor. *Post-hoc* comparisons were further performed to determine longitudinal changes over time within group and differences between groups at each visit time. Abbreviations: hOc41a, the anterior third of the middle and inferior lateral occipital gyri; Fp2, medial frontopolar area 2; T0, fMRI data at onset; T1, the 1-year follow-up evaluation; T2, the 3-year follow-up evaluation. PD-pRBD^+^, Parkinson's disease (PD) with probable rapid eye movement (REM) sleep behavior disorder (pRBD); PD-pRBD^−^, PD without pRBD; HC, healthy control. Lt, left; Rt, right. ^†^showed significant differences between patients with PD-pRBD^+^ and PD-pRBD^−^; ^‡^showed significant differences between patients with PD-pRBD^+^ and HCs. *p < 0.05 with Bonferroni correction; **p < 0.005 with Bonferroni correction; ***p < 0.001 with Bonferroni correction.

**Figure 5:**
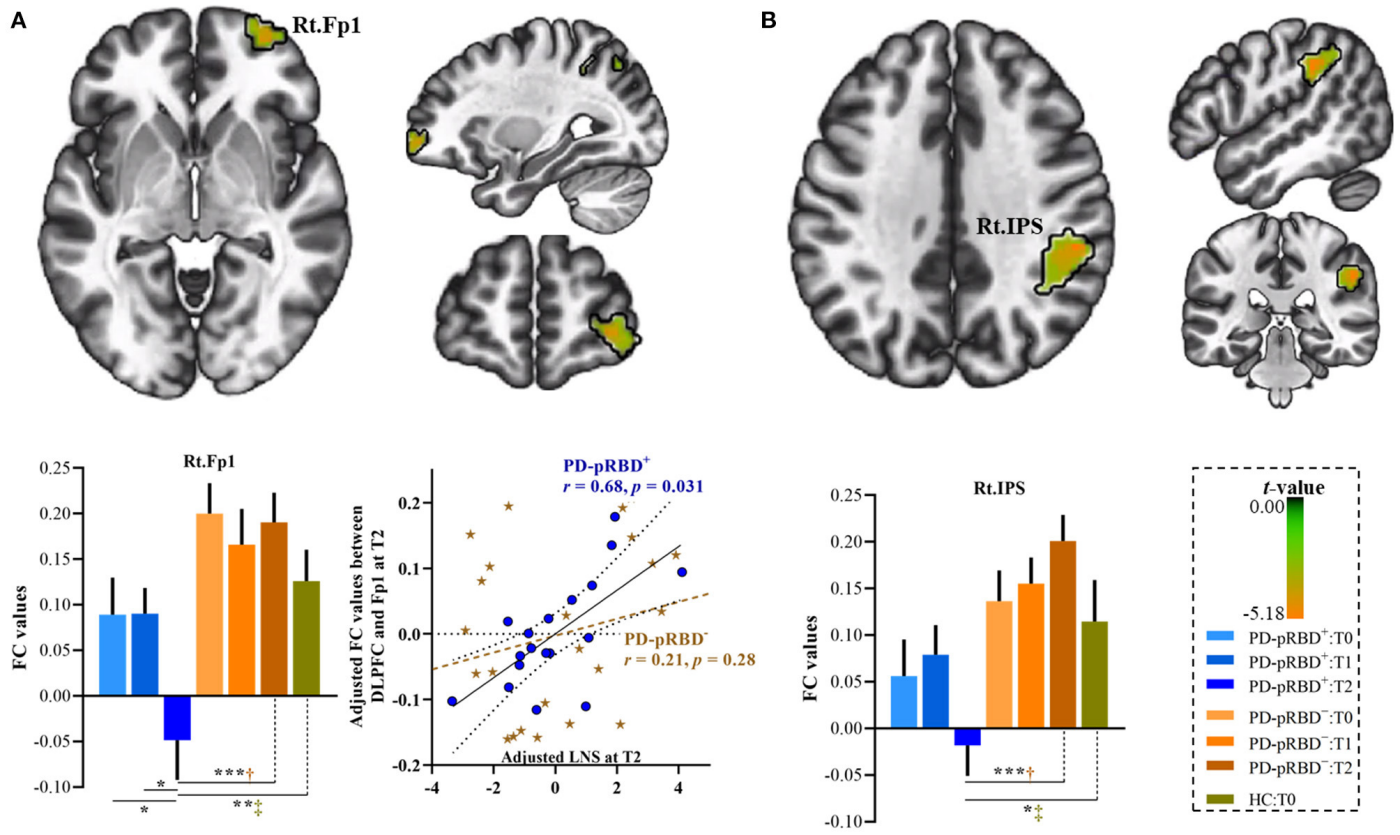
Progressive FC decrease in the left DLPFC in PD-pRBD^+^ compared with PD-pRBD^−^ at 3-year follow-up evaluation. **(A)** Decreased FC in the right Fp1; **(B)** Decreased FC in the right IPS. Bar graph showed the mean extracted FC values in these regions. Scatter plots presented the relationship between FC values and scores of LNS test. The results were thresholded based on an uncorrected voxelwise height threshold of *p* < 0.001 combined with an FWE-corrected clusterwise threshold of *p* < 0.05. Mixed repeated measures analysis of variance was performed to compare the longitudinal changes between PD groups with *visit-time* (i.e., T0, T1, T2) as within-group factor and group (i.e., PD-pRBD^+^ and PD-pRBD^−^) as between-group factor. *Post-hoc* comparisons were further performed to determine longitudinal changes over time within group and differences between groups at each visit time. Raw data for LNS and FC were adjusted for confounding variables of age, sex, education, disease duration, and levodopa dose, as demonstrated in x-axis and y-axis. Fp1, lateral frontopolar area 1; LNS, letter-number sequencing; IPS: intraparietal sulcus; T0, fMRI data at onset; T1, the 1-year follow-up evaluation; T2, the 3-year follow-up evaluation. PD-pRBD^+^, Parkinson's disease (PD) with probable rapid eye movement (REM) sleep behavior disorder (pRBD); PD-pRBD^−^, PD without pRBD; Lt, left; Rt, right. ^†^showed significant differences between patients with PD-pRBD^+^ and PD-pRBD^−^; ^‡^*s*howed significant differences between PD-pRBD^+^ patients and HCs. *p < 0.05 with Bonferroni correction; **p < 0.005 with Bonferroni correction; ***p < 0.001 with Bonferroni correction.

In this work, significantly decreased FC in the left DLPFC was detected in the right medial frontopolar area 2 (Fp2) and the bilateral inferior occipital gyri (hOc4la) in patients with PD-pRBD^+^ compared with patients with PD-pRBD^−^ at baseline (Table 2, Figure 4). Furthermore, significantly decreased FC between the left DLPFC and right lateral frontopolar area (Fp1) and intraparietal sulcus (IPS) were revealed specific to patients with PD-pRBD^+^ compared to PD-pRBD^−^ at the 3-year follow-up evaluation (Table 2, Figure 5). No voxels survived corrected multiple comparisons at the 1-year follow-up evaluation between groups.

**Table 2:** Functional connectivity alterations in the left DLPFC between patients with PD-pRBD^+^ and PD-pRBD^−^ over time.

**Anatomical regions**	**Cluster size (voxel)**	**MNI (x, y, z)**	***T*-value**
**T0: PD-pRBD**^**+**^ **vs. PD-pRBD**^**−**^			
Rt.Fp2	54	(0, 66, 0)	−4.26
Lt.hOc4la	78	(−42, −90, −9)	−4.33
Rt.hOc4la	135	(45, −78, −15)	−4.22
**T1: PD–pRBD**^**+**^ **vs. PD-pRBD**^**−**^			
N/A	N/A	N/A	N/A
**T2: PD-pRBD**^**+**^ **vs. PD–pRBD**^**−**^			
Rt.Fp1	29	(30, 60, −3)	−4.35
Rt.IPS	91	(51, −33, 36)	−5.18

*The results were thresholded based on an uncorrected voxelwise height threshold of p < 0.001 combined with an FWE–corrected clusterwise threshold of p < 0.05. PD-pRBD^+^, Parkinson's disease (PD) with probable rapid eye movement (REM) sleep behavior disorder (pRBD); PD-pRBD^−^, PD without pRBD; DLPFC, dorsolateral prefrontal cortex; Fp1, lateral frontopolar area 1; Fp2, medial frontopolar area 2; hOc4la, anterior third of the middle and inferior lateral occipital gyri; IPS, intraparietal sulcus; T0, fMRI data at onset; T1, the 1-year follow-up evaluation; T2, the 3-year follow-up evaluation; Lt, left; Rt, right; N/A, Not Applicable*.

To further explore the dynamic, progressive changes, connectivity values of these regions were extracted for all patients with PD at all visits, as well as from HCs at baseline for comparison purposes. As shown in Figure 4, it was further found that extracted FC values in this altered region specific to PD-pRBD^+^ at baseline were also significantly decreased compared to the FC values in HCs. It was noted that patients with PD-pRBD^+^ exhibited significant FC recovery in the Fp2 at the 1-year follow-up evaluation and then impaired again at 3-year follow-up evaluation (Figure 4A). Similar patterns were identified in the bilateral occipital cortex (Figure 4B).

At the 3-year follow-up evaluation, in comparison with patients with PD-pRBD^−^, specific alterations in connectivity patterns in the right Fp1 and the IPS in PD-pRBD^+^ patients were also detected when compared to the FC of HCs (Figure 5). Furthermore, within the PD-pRBD^+^ group, patients also exhibited significantly decreased FC in the right Fp1 at the 3-year follow-up evaluation compared to that measured both at baseline and at the 1-year follow-up evaluation (Figure 5A). Correlation analysis further revealed that the connectivity between the left DLPFC and the right Fp1 positively correlated with executive function measured by the LNS test in PD-pRBD^+^ patients (*r* = 0.68, *p* = 0.03), whereas no significant correlation was detected in patients with PD-pRBD^−^ (*r* = 0.21, *p* = 0.28) (Figure 5A).

## Discussion

Our results demonstrated that PD-pRBD^+^ patients exhibited specific FC dysfunction between the DLPFC and frontopolar cortex compared with the FC of patients with PD-pRBD^−^, which in turn was associated with a decline in executive function over time. The present findings provided new information about prefrontal cortex dysfunction specific to patients with PD with RBD during disease progression.

Indeed, RBD in patients with PD has a pervasive influence on both motor and non-motor symptoms (Pagano et al., 2018; Kim et al., 2019; Liu et al., 2020), which provides evidence for the poor prognosis for this disease. Consistently, this work found that patients with PD-pRBD^+^ exhibited a more rapid decline in striatal dopamine transporter binding and cognition, greater daytime sleepiness, and worse disease staging than patients with PD-pRBD^−^ as the disease progressed.

Of note, recent studies have demonstrated the RBD increased risk of cognitive impairment (Boot et al., 2012; Postuma et al., 2019). A recent study has reported no significant differences in cognitive performance between patients with PD-pRBD^+^ and PD-pRBD^−^, whereas cognitive decline is associated with sleep efficiency (Hermann et al., 2020). In this work, we found that patients with PD-pRBD^+^ exhibited a more rapid decline in cognition. One possibility was that lower score at cognitive assessments tests might be caused by sleep disorders, regardless of the presence of PD. However, no significant correlation was detected between cognitive performances and sleep behaviors (RBDSQ score and ESS) in patients with PD-pRBD^+^, which might further eliminate this potential possibility (Table 3). Furthermore, some studies have also reported no significant differences in cognitive performances between patients with PD with and without sleep disorders (Bugalho et al., 2011; Sixel-Döring et al., 2014). The heterogeneity of patients with PD, different measurements of sleep behaviors, and different sample sizes among studies may account for these inconsistencies.

**Table 3:** Correlations between cognitive performance and sleep performance in patients with PD-pRBD^+^ by combining visit times.

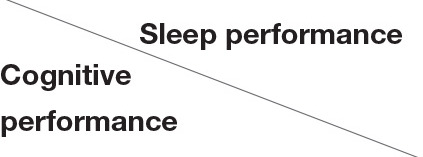	**Statistic values**	**ESS**	**RBDSQ**
MoCA	*r*	−0.19	0.03
	*p*	0.16	0.82
JLO	*r*	−0.14	−0.06
	*p*	0.32	0.65
HVLT-R total recall	*r*	−0.09	0.22
	*p*	0.50	0.12
HVLT-R recognition	*r*	−0.18	0.16
	*p*	0.20	0.24
LNS	*r*	−0.02	0.05
	*p*	0.89	0.72
SFT	*r*	−0.12	−0.13
	*p*	0.40	0.34
SDMT	*r*	−0.25	−0.10
	*p*	0.07	0.49

*PD–pRBD^+^, Parkinson's disease (PD) with probable rapid eye movement (REM) sleep behavior disorder (pRBD); ESS, Epworth Sleepiness Scale; MoCA, Montreal Cognitive Assessment; JLO, Benton Judgment of Line Orientation; HVLT–R, Hopkins Verbal Learning TestRevised; LNS, Letter-Number Sequencing; SFT, semantic fluency test; SDMT, Symbol Digit Modalities Test; RBDSQ, RBD Screening Questionnaire*.

Functional neuroimaging studies have demonstrated the essential role of the DLPFC in executive function (Collette and Van der Linden, 2002; Yang et al., 2009; Jia et al., 2011, 2015b; Liang et al., 2014, 2016). RBD in PD affects underlying higher-level cognitive processes, such as executive function measured by LNS tasks (Lerche and Brockmann, 2018; Hanuška et al., 2019). Accordingly, a recent study has also demonstrated decreased brain activity in the DLPFC in PD-RBD^+^ (Verstraeten and Cluydts, 2004; Ehgoetz Martens et al., 2020).

Importantly, the frontopolar cortex is involved in the most abstract level of executive control for information integration to coordinate dorsolateral functions, subsequently optimizing task performance (Burgess et al., 2000; Fletcher and Henson, 2001; Rogers et al., 2004). In this work, disrupted connectivity between the DLPFC frontopolar cortex was detected specifically in patients with PD-pRBD^+^ compared with PD-pRBD^−^. More specifically, altered connectivity in the Fp2 exhibited an inverse U-shape during the disease progression in patients with PD-pRBD^+^. Actually, increased brain activity in frontal cortex in PD-pRBD^+^ has been reported (Li et al., 2017), possibly due to the upregulated frontal dopamine level to compensate the low dopamine levels in the striatum (Cools et al., 2010). In line with this view, in this work, significant decrease of striatal binding ratios has been identified in PD-pRBD^+^. On this basis, the increased brain activity in prefrontal cortex might reflect compensation for basal ganglia dysfunction at 1-year follow-up in patients with PD-pRBD^+^, whereas, the substantial increase of the LEDD and decrease of striatal dopamine at 3-year follow-up in patients with PD-pRBD^+^ might suggest the loss of compensation during disease progression (Vijayraghavan et al., 2007; Williams-Gray et al., 2007). Of note, alteration in the medial part of frontal pole (Fp2) precedes the lateral area (Fp1) that was observed only at the 3-year follow-up assessment, whereas in the PD-pRBD^−^ group, there is no significant connectivity alteration between the DLPFC and frontopolar cortex over time. Furthermore, correlation analysis suggested that the underlying impaired FC may be responsible for the deficits in executive functions in this disease. Alternatively, it has been argued that dysconnectivity in the frontopolar cortex may be not due to sleep disturbances but to dopaminergic denervation during disease progression, as suggested previously (Kim et al., 2020). However, this possibility was eliminated to some extent, as the results remained after adjusting for the levodopa dose and striatal binding ratios.

Besides, we found that patients with PD-pRBD^+^ exhibited significantly disrupted connectivity between the left DLPFC and right IPS at the 3-year follow-up evaluation. In particular, the IPS plays an important role in executive function such as mental representation and spatial processing (Husain and Nachev, 2007; Gillebert et al., 2011). Note that the frontal–intraparietal network is involved in the development of visuo-spatial working memory (Klingberg, 2006; Jia et al., 2015b); thus the activation of IPS is often associated with simultaneous activity in the dorsolateral frontal lobe (Corbetta and Shulman, 2002; Husain and Rorden, 2003). Moreover, sleep deprivation study has reported that fronto–parietal activation decreases in response to a working memory task (Lythe et al., 2012), which supports the disrupted connectivity between the left DLPFC and right IPS in patients with PD-pRBD^+^. Meanwhile, an increasing number of studies have shown that patients with PD have a high risk of visual hallucination (Stephenson et al., 2010) and patients with RBD are also characterized by the presence of impairment in visuospatial ability (Plomhause et al., 2014). Recent data suggest that dorsal pathway into the parietal cortex and prefrontal cortex involved with visually guided movement and attention control (Gilbert and Li, 2013) is in line with the disrupted connectivity between the left DLPFC and the bilateral occipital cortex in patients with PD-pRBD^+^ at baseline.

In addition, it is noteworthy that thalamostriatal abnormalities in patients with PD have been reported in our recent studies, both structurally (Jia et al., 2015a) and functionally (Jia et al., 2018, 2020). Consistently, GMV alterations associated with patients with PD over time were also identified in the caudate and thalamus. However, no significant regional brain volume differences were identified between the two PD groups in line with a previous study (García-Lorenzo et al., 2013). On this basis, the present findings of disrupted prefrontal cortex functional connectivity were less likely confounded by the structural changes in PD.

### Methodological Considerations

Although our study was strengthened by longitudinal data, several methodological limitations in this work should be acknowledged. The sample size of 46 patients with PD (i.e., 18 PD-pRBD^+^ and 28 PD-pRBD^−^) in this work approximately meets the requirement of 20 samples, as recommended previously for reliability of results based on fMRI (Thirion et al., 2007). In addition, the current results were reported with a strict threshold corrected for multiple comparisons as recommended by Woo et al. (2014) to reduce false positives, and we are reasonably confident about the observed significant effects. However, no voxel survived corrected multiple comparisons at the 1-year follow-up evaluation between groups, suggesting that alterations with small effect size requires a relatively large sample size. Meanwhile, although the RBDSQ has been validated to screen for RBD in patients with PD, the lack of polysomnography may limit the extension of the current findings. Furthermore, interaction analysis including HCs would help clarify more specific changes associated with RBD in patients with PD to rule out the effect of normal aging over time. Thus, further studies are required to carry out more objective measures of RBD and include longitudinal fMRI data of HCs.

## Conclusions

In conclusion, this longitudinal work explored the neurobiological mechanisms associated with sleep disturbances in patients with PD during disease progression. Our results suggest that progressive prefrontal cortex dysfunction in early PD with RBD might be an effective subtype-specific biomarker of the neurodegenerative progression, which further advances the present understanding of cortical dysfunction and sheds light on the neuropathological mechanisms and new therapeutic strategy associated with RBD in this disease.

## Data Availability Statement

Data used in the preparation of this article were obtained from PPMI database (www.ppmi-info.org/data). The original contributions presented in the study are included in the article/supplementary material, further inquiries can be directed to the corresponding authors.

## Ethics Statement

The study was approved by the institutional review board at each PPMI site. The patients/participants provided their written informed consent to participate in this study. Written informed consent was obtained from the individual(s) for the publication of any potentially identifiable images or data included in this article.

## Author Contributions

XJ, JW, and QY contributed to design the study. XJ, WF, ZW, YLiu, YLi, and HaL contributed to acquire and analyze the data. XJ, WF, ZW, YLi, HuL, TM, JW, and QY contributed to interpret the findings and draft the manuscript. XJ, TM, JW, and QY contributed to revise the manuscript. All authors contributed to the article and approved the submitted version.

## Funding

This work was supported by grants from the National Natural Science Foundation of China (62076169), Beijing Hospitals Authority Youth Program (QML20200304), and Basic research foundation of Shenzhen Science and Technology Stable Support Plan (GXWD20201230155427003-20200822115709001).

## Conflict of Interest

The authors declare that the research was conducted in the absence of any commercial or financial relationships that could be construed as a potential conflict of interest.

## Publisher's Note

All claims expressed in this article are solely those of the authors and do not necessarily represent those of their affiliated organizations, or those of the publisher, the editors and the reviewers. Any product that may be evaluated in this article, or claim that may be made by its manufacturer, is not guaranteed or endorsed by the publisher.
